# ELECTROCONVULSIVE THERAPY FOR AGITATION IN LEWY BODIES DEMENTIA

**DOI:** 10.1192/j.eurpsy.2023.2167

**Published:** 2023-07-19

**Authors:** C. Echeverria, J. Libuy, J. Alarcón, J. Rodriguez

**Affiliations:** 1 Resident in Psychiatry, Universidad de los Andes; 2 Adjunct Instructor, Pontifica Universidad Católica de Chile; 3 Psychiatry, Hospital Clínico Metropolitano Dra. Eloísa Díaz, Santiago, Chile

## Abstract

**Introduction:**

Dementia with Lewy Bodies (DLB) is a primary degenerative dementing syndrome characterized by visual hallucinations, fluctuation in cognition, depressive symptoms and parkinsonism. Literature has shown the utility of electroconvulsive therapy (ECT) in demented patients regarding depressive symptoms and agitation. Nevertheless, the majority of cases described include patients with vascular dementia and Alzheimer’s disease. There are no cases informed concerning ECT in DLB patients with agitation and aggressive behaviors.

**Objectives:**

Evaluate the impact of electroconvulsive therapy (ECT) for agitation in a patient with diagnosis of Lewy Bodies Dementia (DLB).

**Methods:**

Case report. 68-year-old male, with no prior neuropsychiatric history, was present for psychiatric evaluation for 5 year history of progressive dementia with fluctuations in cognition, complex visual hallucinations, delusional beliefs, depressive mood, anhedonia, irritability, associated to parkinsonism and increasing autoaggressive behaviors and agitation.

An extensive neurologic workup including neuroimaging, EEG and laboratory studies failed to reveal a specific etiology. Neuropsychological testing reveals frontal, attentional, and visuospatial dysfunction. A presumptive diagnosis of DLB was made.

Medication trials including donepezil, memantine, lamotrigine, sertraline, quetiapine, risperidone and melatonin failed to manage his depressive, psychotic and behavioral disturbances.

**Results:**

Considering past medication failures and prominent behavioral disturbances family consented for an acute course of ECT.

Initial acute phase consisted of 6 sessions of right unilateral, brief pulse width (0.3 ms) ECT tri-weekly utilizing Mecta Spectrum. Anesthesia was induced with propofol, and received succinylcholine for muscle relaxation. Initial charge was 115 mC (6x seizure threshold), then raised to 192 mC. Seizure duration averaged in 22 seconds. No adverse reactions reported.

Clinical outcomes were measured with the CGI-Efficacy Index. Pre-ECT CGI-SI score was 6 (severely ill) and post-ECT CGI-I was 3 (minimally improved).

**Conclusions:**

Mood and behavioral disturbances are a frequent primary motive consultations in DLB patients. The treatment is challenging due to the sensitivity to antidopaminergic medications evidenced in this type of patients. This case suggests that ECT has an impact in the treatment of agitation and aggression in DLB patients, although further investigation is needed.

**Disclosure of Interest:**

None Declared

